# Farm Animal Contact as Risk Factor for Transmission of Bovine-associated *Salmonella* Subtypes

**DOI:** 10.3201/eid1812.110831

**Published:** 2012-12

**Authors:** Kevin J. Cummings, Lorin D. Warnick, Margaret A. Davis, Kaye Eckmann, Yrjö T. Gröhn, Karin Hoelzer, Kathryn MacDonald, Timothy P. Root, Julie D. Siler, Suzanne M. McGuire, Martin Wiedmann, Emily M. Wright, Shelley M. Zansky, Thomas E. Besser

**Affiliations:** Author affiliations: Texas A&M University, College Station, Texas, USA (K.J. Cummings);; Cornell University, Ithaca, New York, USA (K.J. Cummings, L.D. Warnick, Y.T. Gröhn, K. Hoelzer, J.D. Siler, M. Wiedmann, E.M. Wright);; Washington State University, Pullman, Washington, USA (M.A. Davis, T.E. Besser);; Washington State Department of Health, Olympia, Washington, USA (K. Eckmann, K. MacDonald);; New York State Department of Health, Albany, New York, USA (T.P. Root, S.M. McGuire, S.M. Zansky)

**Keywords:** Salmonella, salmonellosis, subtypes, infectious disease transmission, risk factors, case-control study, public health, bacteria, farm animal contact, cattle, bovine

## Abstract

Salmonellosis prevention should focus on safe animal contact as well as food safety.

## Medscape CME Activity

Medscape, LLC is pleased to provide online continuing medical education (CME) for this journal article, allowing clinicians the opportunity to earn CME credit. 

This activity has been planned and implemented in accordance with the Essential Areas and policies of the Accreditation Council for Continuing Medical Education through the joint sponsorship of Medscape, LLC and Emerging Infectious Diseases. Medscape, LLC is accredited by the ACCME to provide continuing medical education for physicians.

Medscape, LLC designates this Journal-based CME activity for a maximum of 1 *AMA PRA Category 1 Credit(s)*^TM^. Physicians should claim only the credit commensurate with the extent of their participation in the activity.

All other clinicians completing this activity will be issued a certificate of participation. To participate in this journal CME activity: (1) review the learning objectives and author disclosures; (2) study the education content; (3) take the post-test with a 70% minimum passing score and complete the evaluation at www.medscape.org/journal/eid; (4) view/print certificate.

**Release date: November 14, 2012; Expiration date: November 14, 2013**

## Learning Objectives

Upon completion of this activity, participants will be able to:

- Analyze the epidemiology of salmonellosis.
- Distinguish broad characteristics of patients with salmonellosis in the current study.
- Assess risk factors for bovine-associated salmonellosis in the current study.

## CME Editor

**P. Lynne Stockton, VMD, MS, ELS(D), **Technical Writer/Editor, *Emerging Infectious Diseases. Disclosure: P. Lynne Stockton, VMD, MS, ELS(D), has disclosed no relevant financial relationships.*

## CME Author

**Charles P. Vega, MD, **Health Sciences Clinical Professor; Residency Director, Department of Family Medicine, University of California, Irvine. *Disclosure: Charles P. Vega, MD, has disclosed no relevant financial relationships.*

## Authors

*Disclosures:* ***Kevin J. Cummings, DVM, PhD*** *;* ***Margaret A. Davis, DVM, MPH, PhD*** *;* ***M. Kaye Eckmann, BS*** *;* ***Yrjö T. Gröhn, DVM, PhD*** *;* ***Karin Hoelzer, PhD*** *;* ***Kathryn MacDonald, PhD, RN*** *;* ***Timothy P. Root*** *;* ***Julie D. Siler*** *;* ***Suzanne M. McGuire, RN, BSN, CCTC*** *;* ***Emily M. Wright*** *,* ***BS*** *;* ***Shelley M. Zansky, PhD*** *; and* ***Thomas E. Besser, DVM, PhD, DACVM*** *, have disclosed no relevant financial relationships.* ***Lorin D. Warnick, DVM, PhD*** *, has disclosed the following relevant financial relationships: served as an advisor or consultant for Pfizer Animal Health.* ***Martin Wiedmann, DVM*** *, has disclosed the following relevant financial relationships: served as an advisor or consultant for Roka; owns stock, stock options, or bonds from Neogen, Sample6 Technologies.*
