# Supplementary material for: Farm Animal Contact as Risk Factor for Transmission of Bovine-associated Salmonella Subtypes
Source: Emerg Infect Dis. 2012 Dec;18(12):1929–36. doi: 10.3201/eid1812.110831 (PMC3557873; doi:10.3201/eid1812.110831)
Supplement: Related texts

# Research article: Farm Animal Contact as Risk Factor for Transmission of Bovine-associated *Salmonella* Subtypes

**Authors:** Kevin J. Cummings, Lorin D. Warnick, Margaret A. Davis, Kaye Eckmann, Yrjö T. Gröhn, Karin Hoelzer, Kathryn MacDonald, Timothy P. Root, Julie D. Siler, Suzanne M. McGuire, Martin Wiedmann, Emily M. Wright, Shelley M. Zansky, Thomas E. Besser

**Affiliations:** Author affiliations: Texas A&M University, College Station, Texas, USA (K.J. Cummings);; Cornell University, Ithaca, New York, USA (K.J. Cummings, L.D. Warnick, Y.T. Gröhn, K. Hoelzer, J.D. Siler, M. Wiedmann, E.M. Wright);; Washington State University, Pullman, Washington, USA (M.A. Davis, T.E. Besser);; Washington State Department of Health, Olympia, Washington, USA (K. Eckmann, K. MacDonald);; New York State Department of Health, Albany, New York, USA (T.P. Root, S.M. McGuire, S.M. Zansky)

Salmonellosis is usually associated with foodborne transmission. To identify risk from animal contact, we compared animal exposures of case-patients infected with bovine-associated *Salmonella* subtypes with those of control-patients infected with non-bovine–associated subtypes. We used data collected in New York and Washington, USA, from March 1, 2008, through March 1, 2010. Contact with farm animals during the 5 days before illness onset was significantly associated with being a case-patient (odds ratio 3.2, p = 0.0008), after consumption of undercooked ground beef and unpasteurized milk were controlled for. Contact with cattle specifically was also significantly associated with being a case-patient (odds ratio 7.4, p = 0.0002), after food exposures were controlled for. More cases of bovine-associated salmonellosis in humans might result from direct contact with cattle, as opposed to ingestion of foods of bovine origin, than previously recognized. Efforts to control salmonellosis should include a focus on transmission routes other than foodborne.

*Salmonella* *enterica* remains a formidable public health challenge, resulting in ≈1.2 million illnesses and 400 deaths annually in the United States alone (*1*). Disease manifestations include diarrhea, fever, anorexia, abdominal pain, vomiting, and malaise. Although clinical disease generally resolves within 3–7 days, *Salmonella* spp. can also produce potentially fatal invasive infections. The incidence of human salmonellosis has not declined over the past 15 years and is significantly higher than it was during 2006–2008 (*2*). An estimated 94% of *Salmonella* infections are foodborne (*1*); common sources include undercooked eggs, poultry, beef, and pork; unpasteurized dairy products; and raw vegetables (*3**–**7*). Although some studies have shown that direct contact with infected animals is a risk factor for salmonellosis (*8**,**9*), the foodborne route is still regarded as the primary transmission route.

Dairy cattle are considered a key source of several *Salmonella* serovars that are a threat to human health, including multidrug-resistant *S*. *enterica* serovar Newport and *S*. *enterica* serovar Typhimurium (*8**–**11*). Foodborne transmission can occur through fecal contamination of beef carcasses at the time of slaughter (*12*) or through contamination of crops, either by manure used as fertilizer or by manure-contaminated irrigation water (*13*). Milk and other dairy products pose less of a public health threat because of commercial pasteurization, although consumption of unpasteurized dairy products persists. Infection by direct contact is an occupational risk for dairy farm workers and veterinarians. The most recent National Animal Health Monitoring System Dairy Study reports that there were >75,000 dairy operations in the United States in 2006, and the American Veterinary Medical Association reports that there were >5,000 veterinarians engaged either predominantly or exclusively in food animal practice as of 2010. Persons who interact with dairy cattle in public settings, such as open farms, petting zoos, and county or state fairs, are also at risk for salmonellosis through direct exposure (*8**,**9**,**14**,**15*).

Our objective was to identify significant risk factors for salmonellosis caused by bovine-associated *Salmonella* subtypes (including those within the Newport and Typhimurium serovars) by using the case–case study design (*16*). We specifically evaluated the role of direct animal contact as a potential route of transmission.

## Materials and Methods

### Study Population

This case–case study was conducted by using culture-confirmed human salmonellosis cases reported in targeted geographic areas in the states of New York and Washington, USA. Specimens were collected from March 1, 2008, through March 1, 2010.

#### New York State

Public Health Law in New York requires laboratories and physicians to report all salmonellosis cases to local health departments and to submit isolates to the New York State Department of Health (NYSDOH) Wadsworth Center Public Health Laboratory for diagnostic confirmation and speciation. The local health departments submit all case information, including laboratory data and questionnaire results, to the NYSDOH by a secure electronic data collection system. Within the FoodNet (www.cdc.gov/foodnet/) catchment area of the Centers for Disease Control and Prevention (CDC) Emerging Infections Program, surveillance officers actively monitor clinical microbiology laboratories and contact local health departments to ascertain all laboratory-confirmed salmonellosis cases, and they review case reports for accuracy and completeness. This catchment area includes 34 counties in the Albany, Buffalo, and Rochester areas of New York, representing ≈4.3 million residents (22% of the total state population).

#### Washington State

As in New York, salmonellosis is reportable in the state of Washington, and clinical laboratories are required to submit all isolates to the Washington State Department of Health (WSDOH) Public Health Laboratories for further characterization. County health departments submit all case information to the WSDOH Communicable Disease Epidemiology Unit. The 6 participating counties in Washington were King, Pierce, Snohomish, Spokane, Whatcom, and Yakima. These included 3 of the most populous counties (King, Pierce, and Snohomish) and 2 counties with the highest concentrations of dairy cattle (Whatcom and Yakima) in Washington. Spokane County comprises an urban population in addition to rural and farming communities. The 6 participating counties represent ≈4.3 million residents (65% of the total state population).

### Laboratory Methods

In New York, serotyping and pulsed-field gel electrophoresis (PFGE) were performed on all *Salmonella* FoodNet isolates received by the NYSDOH during the study period. Typing data were forwarded to Cornell University (Ithaca, NY, USA) for PFGE pattern comparison by BioNumerics software (Applied Maths Inc., Austin, TX, USA). Confirmed *Salmonella* isolates of bovine origin, obtained either from clinical samples submitted to the Cornell University Animal Health Diagnostic Center or from field study samples collected from clinically ill and asymptomatic dairy cattle, were sent to the US Department of Agriculture, Animal and Plant Health Inspection Service, National Veterinary Services Laboratories (Ames, IA, USA) for serotyping by standard protocols. PFGE subtyping of bovine isolates was performed in the Food Science Laboratory at Cornell University. The standard CDC PulseNet protocol (*17*) was used for subtyping all study isolates.

In Washington, serotyping and PFGE were performed on all human clinical isolates submitted to the WSDOH as described for New York. Bovine isolates, obtained either from clinical samples submitted to the Washington Animal Disease Diagnostic Laboratory or from samples collected during dairy cattle field studies, were also sent to the National Veterinary Services Laboratories for serotyping. PFGE subtyping of bovine isolates was performed at Washington State University, again by using the standard CDC PulseNet protocol.

### Questionnaire

As part of the routine investigation of foodborne *Salmonella* infections, trained interviewers from local health departments in both states administered a standardized questionnaire to each patient by telephone. The NYSDOH *Salmonella* questionnaire was adapted from a previous version used for investigating all cases of foodborne infection. The standard WSDOH questionnaire was supplemented with an additional set of questions to ensure completeness of exposure data collection and to better align Washington data with New York data. Patient identification data were removed from each dataset before being transferred to the university research group in the respective state. Data collected in each state included demographic information, clinical features, and exposure history during the 5 days before disease onset. Exposure data included animal contacts, food history, food hygiene practices, water use for drinking and recreation, health care or daycare exposures, and travel history. After data collection had ended, both datasets were compiled at Cornell University for analysis.

### Case-Patients and Control-Patients

Eligible cases included *Salmonella* spp.–positive patients from the NYS FoodNet catchment area and the 6 participating Washington counties that were identified during the study period. Patients were excluded if they were associated with an obvious outbreak (as noted by the state health departments) or if they had a typhoidal *Salmonella* infection (either Typhi or Paratyphi A). For the case–control analysis, case-patients were defined as patients infected with *Salmonella* isolates that matched contemporary bovine isolates from the respective state by serovar and PFGE pattern. Control-patients were defined as patients infected with *Salmonella* isolates that were not associated with cattle, according to those criteria. Specifically, all patients infected with *S*. *enterica* serovar Dublin were classified as case-patients because this serovar is host-adapted to cattle (*18*). Patients infected with 6 other serovars (Newport, Typhimurium, Infantis, 4,5,12:i:–, Agona, and Montevideo) were classified as potential case-patients because of the importance of these serovars in bovine and human hosts.

According to a recent comprehensive study on the incidence of salmonellosis among dairy herds in New York and other northeastern states, the first 5 serovars just mentioned were among the leading serovars shed by dairy cattle with clinical *Salmonella* infections (*19*), and Montevideo is consistently one of the most prevalent serovars shed by asymptomatic cattle (*20*). All 6 are among the top 20 serovars isolated from human patients with laboratory-confirmed salmonellosis in the United States (*21*). For patients infected with one of the aforementioned serovars, PFGE patterns from the human isolates were compared with those from cattle. To be considered bovine associated, an isolate had to have a PFGE pattern indistinguishable from that of isolates obtained from >2 cattle in the same state from March 1, 2007, through March 1, 2010; patients infected with such isolates were thus classified as case-patients. Human isolates that differed from the most similar bovine isolate by 1–3 visible bands were excluded from the analysis, as were human isolates with a PFGE pattern matching that of just 1 bovine animal. Patients infected with isolates that differed from the most similar bovine isolate by >4 visible bands were classified as control-patients.

Patients infected with *Salmonella* serovars other than those previously listed were classified as control-patients if the serovar was not detected in cattle in the same state during that time frame. If the serovar was detected in cattle, the human isolate had to differ from the most similar bovine isolate by >4 visible bands in order for that patient to be considered a control-patient; otherwise, the human isolate was excluded from the analysis. A total of 422 bovine isolates from New York and 447 bovine isolates from Washington were used for PFGE pattern comparison.

### Data Analysis

Data were imported into a commercially available statistical software program (SAS, version 9.2; SAS Institute Inc., Cary, NC, USA) for variable coding and analysis. Age was converted into a categorical variable (<5, 5–12, 13–20, 21–40, 41–60, and >60 years of age). Animal, food, and other exposures were analyzed as dichotomous variables (yes/no). The variables “farm animal contact” and “bovine contact” were created to most effectively capture data from 2 state health department questionnaires that were not identical. In the New York dataset, farm animal contact was considered “yes” if the patient reported an occupation of animal farming or a history of farm animal contact; bovine contact was considered “yes” if the patient specified cattle as the type of farm animal. In the Washington dataset, farm animal contact was considered “yes” if the patient reported a history of living or working on a dairy or other farm type or reported a history of contact with cattle, sheep, goats, horses, or pigs; bovine contact was considered “yes” if the patient specified cattle as the type of farm animal.

Analysis was performed to compare exposures between case-patients and control-patients. Univariable descriptive analysis was performed on all explanatory variables. Bivariable analysis with the χ^2^ test was used to determine whether each variable was independently associated with case or control status. Multivariable logistic regression models were used to identify risk factors for infection with bovine-associated subtypes; case or control status was used as the dichotomous outcome variable. Initial selection of variables was based on the bivariable analysis screening (p<0.25), and a backward elimination approach was used to identify a final multivariable model; values of p<0.05 were considered significant. Relevant 2-way interaction terms (involving exposure variables retained in the final model, demographic variables, and state) were also investigated for significance within each model. Consumption of undercooked ground beef and unpasteurized milk in the 5 days before disease onset were included in each model as potential confounders. The population attributable fraction (PAF), defined as the proportion of disease in a population that can be attributed to a given exposure, was calculated for variables retained in each model by using the formula PAF = P(OR_adj_ – 1)/OR_adj_ (where P = the proportion of case-patients exposed to the risk factor and OR_adj_ = the adjusted odds ratio for that factor) (*22*).

## Results

From March 1, 2008, though March 1, 2010, the NYSDOH received nontyphoidal *Salmonella* isolates from 835 patients within the NYS FoodNet catchment area. According to our criteria, 40 (4.8%) of these were classified as case-patients and 356 (42.6%) as control-patients. Among case-patients, 20 (50.0%) were female; among control-patients, 215 (60.4%) were female. The median age among case-patients was 31.5 years, whereas that among control-patients was 31 years. Typhimurium was the most common serovar among case-patients (67.5%), and Enteritidis and Typhimurium were equally predominant among control-patients (10.1%; Table 1).

**Table 1:** Distribution of *Salmonella *serovars among 835 patients, New York, USA, March 1, 2008–March 1, 2010

Serovar	No. (%)
Case-patients, n = 40	
Typhimurium	27 (67.5)
Dublin	7 (17.5)
Newport	5 (12.5)
Infantis	1 (2.5)
Control-patients, n = 356	
Enteritidis	36 (10.1)
Typhimurium	36 (10.1)
Heidelberg	32 (9.0)
Newport	21 (5.9)
Braenderup	16 (4.5)
Javiana	14 (3.9)
Saintpaul	13 (3.7)
Hadar	12 (3.4)
B,5:i:–	11 (3.1)
Muenchen	10 (2.8)
Agona	8 (2.2)
Berta	8 (2.2)
Paratyphi B var. L-tartrate+	8 (2.2)
B,5:b:–	7 (2.0)
Poona	7 (2.0)
Stanley	7 (2.0)
Hartford	6 (1.7)
Miami	6 (1.7)
Montevideo	6 (1.7)
Schwarzengrund	5 (1.4)
Bovismorbificans	4 (1.1)
Derby	4 (1.1)
Ealing	4 (1.1)
Manhattan	4 (1.1)
Mississippi	4 (1.1)
Other serovars	67 (18.8)
Excluded patients, n = 439	
Enteritidis	195 (44.4)
Typhimurium	99 (22.6)
Thompson	22 (5.0)
Oranienburg	18 (4.1)
Newport	14 (3.2)
Montevideo	8 (1.8)
Infantis	7 (1.6)
Tennessee	7 (1.6)
Panama	6 (1.4)
Other serovars	46 (10.5)
Not typed	17 (3.9)

During the study period, 562 patients with nontyphoidal salmonellosis were identified in the 6 participating Washington counties. According to our criteria, 87 (15.5%) of these were classified as case-patients and 428 (76.2%) as control-patients. Among case-patients, 53 (60.9%) were female; among control-patients, 229 (53.5%) were female. The median age among case-patients was 28 years, whereas that among control-patients was 33 years. The most common serovar among case-patients was Typhimurium (51.7%), and the most common serovar among control-patients was Enteritidis (40.4%; Table 2).

**Table 2:** Distribution of *Salmonella* serovars among 562 patients, Washington, USA, March 1, 2008–March 1, 2010

Serovar	No. (%)
Case-patients, n = 87	
Typhimurium	45 (51.7)
Montevideo	14 (16.1)
Newport	13 (14.9)
4,5,12:i:–	9 (10.3)
Dublin	5 (5.7)
Infantis	1 (1.1)
Control-patients, n = 428	
Enteritidis	173 (40.4)
Typhimurium	29 (6.8)
Paratyphi B var. L-tartrate+	23 (5.4)
Javiana	11 (2.6)
Montevideo	11 (2.6)
Stanley	10 (2.3)
Braenderup	9 (2.1)
Litchfield	9 (2.1)
Thompson	9 (2.1)
4,5,12:i:–	8 (1.9)
Heidelberg	8 (1.9)
Muenchen	7 (1.6)
Senftenberg	7 (1.6)
Virchow	7 (1.6)
Agona	6 (1.4)
Potsdam	6 (1.4)
Saintpaul	6 (1.4)
Oranienburg	5 (1.2)
Other serovars	84 (19.6)
Excluded patients, n = 47	
Typhimurium	7 (14.9)
Heidelberg	6 (12.8)
Montevideo	6 (12.8)
Brandenburg	4 (8.5)
Oranienburg	3 (6.4)
Saintpaul	3 (6.4)
4,5,12:i:-	2 (4.3)
Infantis	2 (4.3)
Newport	2 (4.3)
Panama	2 (4.3)
1,4,5,12:i:-	1 (2.1)
Anatum	1 (2.1)
Enteritidis	1 (2.1)
Hadar	1 (2.1)
Mbandaka	1 (2.1)
Oslo	1 (2.1)
Sandiego	1 (2.1)
Uganda	1 (2.1)
Not typed	2 (4.3)

The datasets from each state were combined to yield a total of 127 case-patients and 784 control-patients. Bivariable analysis indicated that more case-patients (11.0%) than control-patients (3.8%) reported a history of farm animal contact during the 5 days before disease onset (p = 0.0004). More case-patients (6.3%) than control-patients (1.0%) also reported a specific history of bovine contact during the 5 days before illness (p<0.0001). Attendance at an open farm/petting zoo/fair was more common (p = 0.05) among case-patients (11.8%) than control-patients (7.0%), and more case-patients (12.6%) than control-patients (6.5%) reported a history of contact with animal manure (p = 0.01). Fewer case-patients (3.1%) than control-patients (13.9%) reported a history of international travel before illness (p = 0.0006). Case-patients and control-patients did not differ significantly with respect to sex, age group, eating undercooked ground beef, or drinking unpasteurized milk.

Multivariable logistic regression analysis showed that a history of farm animal contact during the 5 days before disease onset was significantly associated with being a case-patient (odds ratio [OR] 3.2, 95% CI 1.6–6.4, p = 0.0008), after consumption of undercooked ground beef and unpasteurized milk was accounted for (Table 3). A specific history of bovine contact during the 5 days before illness was also significantly associated with being a case-patient (OR, 7.4, 95% CI 2.6–20.9, p = 0.0002), according to estimates from a separate logistic regression model that also controlled for those food exposures (Table 4). International travel was negatively associated with being a case-patient in each of the models. No significant interaction was found between farm animal/bovine contact and state, sex, or age group in the respective models. The PAF, applied here as the proportion of *Salmonella* infections among the source population of laboratory-confirmed cases that can be attributed to a certain exposure, was calculated to be 7.6% for farm animal contact and 5.4% for bovine contact in particular.

**Table 3:** Association between infection with a bovine-associated *Salmonella *subtype and farm animal contact, New York and Washington, USA, March 1, 2008–March 1, 2010*

Variable	Odds ratio (95% CI)	p value
Farm animal contact	3.2 (1.6–6.4)	0.0008
Undercooked ground beef	1.5 (0.7–3.1)	0.3
Unpasteurized milk	0.5 (0.1–4.2)	0.5
International travel	0.2 (0.1–0.6)	0.002

*Estimated by a logistic regression model.

**Table 4:** Association between infection with a bovine-associated *Salmonella* subtype and bovine contact, New York and Washington, USA, March 1, 2008–March 1, 2010*

Variable	Odds ratio (95% CI)	p value
Bovine contact	7.4 (2.6–20.9)	0.0002
Undercooked ground beef	1.6 (0.8–3.2)	0.2
Unpasteurized milk	0.5 (0.1–5.1)	0.6
International travel	0.2 (0.1–0.5)	0.002

*Estimated by a logistic regression model.

To perform a sensitivity analysis for testing the effect of our strict case definition, we repeated multivariable logistic regression models under 2 extreme scenarios; all potential case-patients that were excluded (because the isolate differed from the most similar bovine isolate by 1–3 visible bands or because its PFGE pattern matched that of just 1 bovine animal) served alternatively as case-patients (scenario 1) and control-patients (scenario 2). The parameter estimates and ORs from these hypothetical models were comparable to those obtained from our original analyses (the ORs under scenarios 1 and 2 were 3.2 and 2.3 for farm animal contact, 6.1 and 4.0 for bovine contact, respectively).

## Discussion

The case–case study design proposed by McCarthy and Giesecke is an adaptation of the conventional case–control approach (*16*). It has been used to study risk factors and clinical features associated with particular subtypes of *Salmonella* spp. (*8**,**23**–**26*), *Campylobacter* spp. (*27**,**28*), and *Clostridium difficile* (*29*). One of its main advantages is the removal of selection bias imposed by the surveillance system; case-patients and control-patients were subjected to the same selection process in order to be detected by a state health department as a laboratory-confirmed case. Another advantage is the negation of recall bias (a form of information bias); because case-patients and control-patients had salmonellosis, their recall of exposures should have been similarly affected by attitudes regarding causation.

A potential limitation of the case–case study design is that the control-patients might not represent the exposure prevalence in the source population on account of the unique exposures that led them to become infected. However, we believe that we addressed this issue by including a diverse array of serovars and PFGE types in the control group, assuming that their associated exposures were presumably also diverse and thus more representative of the total spectrum of exposures associated with nonbovine *Salmonella* strains. Another possible drawback of this study design is that case-patients and control-patients share a certain subset of exposures that pose a risk for *Salmonella* infections in general; such exposures will therefore remain unidentified or at least be underestimated as risk factors. Although this study design precludes the study of general risk factors for salmonellosis, it is useful for investigating exposures that are serovar or subtype specific.

Other studies have found an association between salmonellosis and having previous contact with either cattle or a farm environment (*8**,**9**,**30*). Our study investigated this association with a case–case approach that used a strict case definition. Another strength of this study was the use of sporadic cases of salmonellosis rather than cases associated with outbreaks. Insight regarding the epidemiology of sporadic *Salmonella* infections has traditionally been limited because specific sources of enteric illness are seldom identified when not occurring as part of an outbreak.

Direct contact with dairy cattle or their environment during the 5 days before illness onset was significantly associated with salmonellosis caused by a bovine-matched subtype in New York and Washington. Because there was no interaction between state and the animal contact variables in the models, we concluded that the effect estimate was consistent across the 2 states. The ORs for farm animal contact and specific bovine contact in each state were also similar to those obtained from analysis of the combined dataset (data not shown). In addition, our sensitivity analysis led us to decide that we still would have reached the same conclusions with modified case criteria. These results have important implications for dairy farm workers and their families, veterinarians and veterinary staff, and those who interact with dairy cattle in public settings.

Although attendance at an open farm/petting zoo/fair was not significantly associated with being a case-patient in this study, it is logical to believe that visiting such a facility might increase the risk for salmonellosis, on the basis of our other findings. *S. enterica* is transmitted primarily by the fecal–oral route. Direct contact with the feces of infected cattle can occur through feeding, petting, or otherwise handling them; contaminated clothing or footwear, animal bedding, barriers, or other environmental surfaces can also be sources of infection (*15**,**31*). This threat is underscored by the recent finding that the median duration of fecal *Salmonella* shedding following clinical disease among dairy cattle is 50 days (*32*).

The negative association between recent international travel and salmonellosis caused by a bovine-matched subtype was anticipated. Although travel outside the United States is a well-known risk factor for *Salmonella* infections (*33*) (observed in a significantly higher proportion of control-patients in this study), it would not be expected to have an association with salmonellosis caused specifically by subtypes shared by dairy cattle in New York and Washington.

The percentage of *Salmonella* infections in the United States that are foodborne was recently estimated at 94% (*1*). The results of our statistical analyses suggest that this percentage might be an overestimate, at least for bovine-associated *Salmonella* subtypes, although our PAF results (which also take into account the frequency of exposure) are more consistent with this estimate. It also must be noted that the effect of animal exposure observed in New York and Washington might not be representative of the rest of the country. Nevertheless, more human infections originating from bovine sources might result from direct contact with cattle (as opposed to foods of bovine origin) than previously recognized. Clear evidence for the role of direct farm animal contact as a source of human salmonellosis indicates that it is imperative for *Salmonella* control efforts to include a focus on transmission routes other than foodborne. The efficacy and public health impact of addressing nonfoodborne transmission of *Salmonella* spp. have been demonstrated by studies of direct contact transmission from pet turtles to humans, particularly children. In response to studies that established turtles as an important source of human salmonellosis (*34**,**35*), federal legislation in 1975 prohibited the sale and distribution of turtles <4 inches in carapace length. Still in effect today, this ban coincided with an 18% reduction in *Salmonella* infections among children 1–9 years of age (*36*).

A number of measures can be taken to minimize the likelihood of becoming infected with *Salmonella* spp. from direct contact with farm animals. Washing hands with soap and water is a simple yet highly protective step that can be taken after contact with animals or feces (*15**,**37*). Children <5 years of age, elderly adults, and immunocompromised persons are at increased risk for invasive salmonellosis (*38**,**39*) and thus should pay special attention to hygiene or avoid certain animal contacts altogether. Veterinarians should teach cattle owners and farm employees to wash well after work or before eating, to disinfect boots and equipment, and to keep coveralls out of the house. If treating infected cattle, veterinarians must specifically counsel their clients about the risk for zoonoses. In particular, high-risk groups should avoid contact with infected cattle. Veterinarians should instruct their staff members to protect themselves by using appropriate infection control procedures (*40*), especially if working with livestock. Increased physician awareness of the role of direct farm animal contact in transmitting *Salmonella* spp. and other enteric zoonotic pathogens is likewise needed. Physicians should educate their patients, particularly those at increased risk for severe disease, regarding the potential threat posed by animal contact and the importance of hand hygiene after such contact. Patients with diarrheal illness should be questioned about their exposures to cattle, farm environments, and other animal species. It is also essential that those who operate open farms and other animal exhibits adhere to current guidelines by equipping such areas with handwashing facilities, preventing food and drink in these areas, maintaining an adequate cleaning and disinfection protocol, and providing visitors with educational materials on disease prevention (*37*). In conclusion, prevention of salmonellosis should include a focus on safe animal contact in addition to food safety measures.

## References

[1] Scallan E, Hoekstra RM, Angulo FJ, Tauxe RV, Widdowson MA, Roy SL, Foodborne illness acquired in the United States—major pathogens. Emerg Infect Dis. 2011;17:7–15 .2119284810.3201/eid1701.P11101PMC3375761

[2] Centers for Disease Control and Prevention. Vital signs: incidence and trends of infection with pathogens transmitted commonly through food—Foodborne Diseases Active Surveillance Network, 10 U.S. sites, 1996–2010. MMWR Morb Mortal Wkly Rep. 2011;60:749–55 .21659984

[3] Centers for Disease Control and Prevention. Multistate outbreak of *Salmonella* serotype Typhimurium infections associated with drinking unpasteurized milk—Illinois, Indiana, Ohio, and Tennessee, 2002–2003. MMWR Morb Mortal Wkly Rep. 2003;52:613–5 .12844078

[4] Centers for Disease Control and Prevention. Multistate outbreak of *Salmonella* Typhimurium infections associated with eating ground beef—United States, 2004. MMWR Morb Mortal Wkly Rep. 2006;55:180–2 .16498383

[5] Centers for Disease Control and Prevention. Multistate outbreak of *Salmonella* infections associated with frozen pot pies—United States, 2007. MMWR Morb Mortal Wkly Rep. 2008;57:1277–80 .19037195

[6] Centers for Disease Control and Prevention. Outbreak of multidrug-resistant *Salmonella enterica* serotype Newport infections associated with consumption of unpasteurized Mexican-style aged cheese—Illinois, March 2006–April 2007. MMWR Morb Mortal Wkly Rep. 2008;57:432–5 .18437118

[7] Centers for Disease Control and Prevention. Outbreak of *Salmonella* serotype Saintpaul infections associated with multiple raw produce items—United States, 2008. MMWR Morb Mortal Wkly Rep. 2008;57:929–34 .18756191

[8] Gupta A, Fontana J, Crowe C, Bolstorff B, Stout A, Van Duyne S, Emergence of multidrug-resistant *Salmonella enterica* serotype Newport infections resistant to expanded-spectrum cephalosporins in the United States. J Infect Dis. 2003;188:1707–16 and. 10.1086/37966814639542

[9] Karon AE, Archer JR, Sotir MJ, Monson TA, Kazmierczak JJ. Human multidrug-resistant *Salmonella* Newport infections, Wisconsin, 2003–2005. Emerg Infect Dis. 2007;13:1777–80 and. 10.3201/eid1311.06113818217570PMC3375811

[10] Dechet AM, Scallan E, Gensheimer K, Hoekstra R, Gunderman-King J, Lockett J, Outbreak of multidrug-resistant *Salmonella enterica* serotype Typhimurium definitive type 104 infection linked to commercial ground beef, northeastern United States, 2003–2004. Clin Infect Dis. 2006;42:747–52 and. 10.1086/50032016477547

[11] Varma JK, Marcus R, Stenzel SA, Hanna SS, Gettner S, Anderson BJ, Highly resistant *Salmonella* Newport-MDRAmpC transmitted through the domestic US food supply: a FoodNet case–control study of sporadic *Salmonella* Newport infections, 2002–2003. J Infect Dis. 2006;194:222–30 and. 10.1086/50508416779729

[12] Wells SJ, Fedorka-Cray PJ, Dargatz DA, Ferris K, Green A. Fecal shedding of *Salmonella* spp. by dairy cows on farm and at cull cow markets. J Food Prot. 2001;64:3–11 .1119843710.4315/0362-028x-64.1.3

[13] Hanning IB, Nutt JD, Ricke SC. Salmonellosis outbreaks in the United States due to fresh produce: sources and potential intervention measures. Foodborne Pathog Dis. 2009;6:635–48 and. 10.1089/fpd.2008.023219580447

[14] Bender JB, Shulman SA. Animals in Public Contact subcommittee, National Association of State Public Health Veterinarians. Reports of zoonotic disease outbreaks associated with animal exhibits and availability of recommendations for preventing zoonotic disease transmission from animals to people in such settings. J Am Vet Med Assoc. 2004;224:1105–9 and. 10.2460/javma.2004.224.110515074855

[15] Smith KE, Stenzel SA, Bender JB, Wagstrom E, Soderlund D, Leano FT, Outbreaks of enteric infections caused by multiple pathogens associated with calves at a farm day camp. Pediatr Infect Dis J. 2004;23:1098–104 .15626945

[16] McCarthy N, Giesecke J. Case–case comparisons to study causation of common infectious diseases. Int J Epidemiol. 1999;28:764–8 and. 10.1093/ije/28.4.76410480708

[17] Ribot EM, Fair MA, Gautom R, Cameron DN, Hunter SB, Swaminathan B, Standardization of pulsed-field gel electrophoresis protocols for the subtyping of *Escherichia coli* O157:H7, *Salmonella*, and *Shigella* for PulseNet. Foodborne Pathog Dis. 2006;3:59–67 and. 10.1089/fpd.2006.3.5916602980

[18] Uzzau S, Brown DJ, Wallis T, Rubino S, Leori G, Bernard S, Host adapted serotypes of *Salmonella enterica.* Epidemiol Infect. 2000;125:229–55 and. 10.1017/S095026889900437911117946PMC2869595

[19] Cummings KJ, Warnick LD, Alexander KA, Cripps CJ, Grohn YT, McDonough PL, The incidence of salmonellosis among dairy herds in the northeastern United States. J Dairy Sci. 2009;92:3766–74 and. 10.3168/jds.2009-209319620658PMC7094388

[20] Centers for Epidemiology and Animal Health. National Animal Health Monitoring System (NAHMS) Dairy 2007: *Salmonella* and *Campylobacter* on U.S. dairy operations, 1996–2007. Washington (DC): US Department of Agriculture; 2009.

[21] Centers for Disease Control and Prevention. *Salmonella* surveillance: annual summary, 2006. Atlanta: US Department of Health and Human Services; 2008.

[22] Kleinbaum DG, Kupper LL, Morgenstern H. Epidemiologic research: principles and quantitative methods. Belmont (CA): Lifetime Learning Publications; 1982.

[23] Van Beneden CA, Keene WE, Strang RA, Werker DH, King AS, Mahon B, Multinational outbreak of *Salmonella enterica* serotype Newport infections due to contaminated alfalfa sprouts. JAMA. 1999;281:158–62 and. 10.1001/jama.281.2.1589917119

[24] Kist MJ, Freitag S. Serovar specific risk factors and clinical features of *Salmonella enterica* ssp. *enterica* serovar Enteritidis: a study in south-west Germany. Epidemiol Infect. 2000;124:383–92 and. 10.1017/S095026889900393310982061PMC2810923

[25] Voetsch AC, Poole C, Hedberg CW, Hoekstra RM, Ryder RW, Weber DJ, Analysis of the FoodNet case–control study of sporadic *Salmonella* serotype Enteritidis infections using persons infected with other *Salmonella* serotypes as the comparison group. Epidemiol Infect. 2009;137:408–16 and. 10.1017/S095026880800089718611288

[26] Aiken AM, Lane C, Adak GK. Risk of *Salmonella* infection with exposure to reptiles in England, 2004–2007. Euro Surveill. 2010;15:19581 .20546689

[27] Smith KE, Besser JM, Hedberg CW, Leano FT, Bender JB, Wicklund JH, Quinolone-resistant *Campylobacter jejuni* infections in Minnesota, 1992–1998. N Engl J Med. 1999;340:1525–32 and. 10.1056/NEJM19990520340200110332013

[28] Gillespie IA, O'Brien SJ, Frost JA, Adak GK, Horby P, Swan AV, A case–case comparison of *Campylobacter coli* and *Campylobacter jejuni* infection: a tool for generating hypotheses. Emerg Infect Dis. 2002;8:937–42 and. 10.3201/eid0809.01081712194770PMC2732536

[29] Morgan OW, Rodrigues B, Elston T, Verlander NQ, Brown DF, Brazier J, Clinical severity of *Clostridium difficile* PCR ribotype 027: a case–case study. PLoS One. 2008;3:e1812.10.1371/journal.pone.0001812PMC226554118350149

[30] Besser TE, Goldoft M, Pritchett LC, Khakhria R, Hancock DD, Rice DH, Multiresistant *Salmonella* Typhimurium DT104 infections of humans and domestic animals in the Pacific Northwest of the United States. Epidemiol Infect. 2000;124:193–200 and. 10.1017/S095026889900328310813142PMC2810900

[31] Steinmuller N, Demma L, Bender JB, Eidson M, Angulo FJ. Outbreaks of enteric disease associated with animal contact: not just a foodborne problem anymore. Clin Infect Dis. 2006;43:1596–602 and. 10.1086/50957617109295

[32] Cummings KJ, Warnick LD, Alexander KA, Cripps CJ, Grohn YT, James KL, The duration of fecal *Salmonella* shedding following clinical disease among dairy cattle in the northeastern USA. Prev Vet Med. 2009;92:134–9 and. 10.1016/j.prevetmed.2009.07.00219665243PMC2753704

[33] Johnson LR, Gould LH, Dunn JR, Berkelman R, Mahon BE. FoodNet Travel Working Group. *Salmonella* infections associated with international travel: a Foodborne Diseases Active Surveillance Network (FoodNet) study. Foodborne Pathog Dis. 2011;8:1031–7 and. 10.1089/fpd.2011.085421563923

[34] Altman R, Gorman JC, Bernhardt LL, Goldfield M. Turtle-associated salmonellosis. II. The relationship of pet turtles to salmonellosis in children in New Jersey. Am J Epidemiol. 1972;95:518–20 .506389310.1093/oxfordjournals.aje.a121419

[35] Lamm SH, Taylor A Jr, Gangarosa EJ, Anderson HW, Young W, Clark MH, Turtle-associated salmonellosis. I. An estimation of the magnitude of the problem in the United States, 1970–1971. Am J Epidemiol. 1972;95:511–7 .506389210.1093/oxfordjournals.aje.a121418

[36] Cohen ML, Potter M, Pollard R, Feldman RA. Turtle-associated salmonellosis in the United States. Effect of public health action, 1970 to 1976. JAMA. 1980;243:1247–9 and. 10.1001/jama.1980.033003800270167359680

[37] National Association of State Public Health Veterinarians, Inc., Centers for Disease Control and Prevention. Compendium of measures to prevent disease associated with animals in public settings, 2011: National Association of State Public Health Veterinarians, Inc. MMWR Recomm Rep. 2011;60:1–24 .21546893

[38] Arshad MM, Wilkins MJ, Downes FP, Rahbar MH, Erskine RJ, Boulton ML, Epidemiologic attributes of invasive non-typhoidal *Salmonella* infections in Michigan, 1995–2001. Int J Infect Dis. 2008;12:176–82 and. 10.1016/j.ijid.2007.06.00617889584

[39] Jones TF, Ingram LA, Cieslak PR, Vugia DJ, Tobin-D'Angelo M, Hurd S, Salmonellosis outcomes differ substantially by serotype. J Infect Dis. 2008;198:109–14 and. 10.1086/58882318462137

[40] Scheftel JM, Elchos BL, Cherry B, DeBess EE, Hopkins SG, Levine JF, Compendium of veterinary standard precautions for zoonotic disease prevention in veterinary personnel: National Association of State Public Health Veterinarians, Veterinary Infection Control Committee 2010. J Am Vet Med Assoc. 2010;237:1403–22 and. 10.2460/javma.237.12.140321155680

# Research article: Farm Animal Contact as Risk Factor for Transmission of Bovine-associated *Salmonella* Subtypes

**Authors:** Kevin J. Cummings, Lorin D. Warnick, Margaret A. Davis, Kaye Eckmann, Yrjö T. Gröhn, Karin Hoelzer, Kathryn MacDonald, Timothy P. Root, Julie D. Siler, Suzanne M. McGuire, Martin Wiedmann, Emily M. Wright, Shelley M. Zansky, Thomas E. Besser

**Affiliations:** Author affiliations: Texas A&M University, College Station, Texas, USA (K.J. Cummings);; Cornell University, Ithaca, New York, USA (K.J. Cummings, L.D. Warnick, Y.T. Gröhn, K. Hoelzer, J.D. Siler, M. Wiedmann, E.M. Wright);; Washington State University, Pullman, Washington, USA (M.A. Davis, T.E. Besser);; Washington State Department of Health, Olympia, Washington, USA (K. Eckmann, K. MacDonald);; New York State Department of Health, Albany, New York, USA (T.P. Root, S.M. McGuire, S.M. Zansky)

## Medscape CME Quiz

## Earning CME Credit

To obtain credit, you should first read the journal article. After reading the article, you should be able to answer the following, related, multiple-choice questions. To complete the questions (with a minimum 70% passing score) and earn continuing medical education (CME) credit, please go to www.medscape.org/journal/eid. Credit cannot be obtained for tests completed on paper, although you may use the worksheet below to keep a record of your answers. You must be a registered user on Medscape.org. If you are not registered on Medscape.org, please click on the New Users: Free Registration link on the left hand side of the website to register. Only one answer is correct for each question. Once you successfully answer all post-test questions you will be able to view and/or print your certificate. For questions regarding the content of this activity, contact the accredited provider, CME@medscape.net. For technical assistance, contact CME@webmd.net. American Medical Association’s Physician’s Recognition Award (AMA PRA) credits are accepted in the US as evidence of participation in CME activities. For further information on this award, please refer to http://www.ama-assn.org/ama/pub/category/2922.html. The AMA has determined that physicians not licensed in the US who participate in this CME activity are eligible for *AMA PRA Category 1 Credits*™. Through agreements that the AMA has made with agencies in some countries, AMA PRA credit may be acceptable as evidence of participation in CME activities. If you are not licensed in the US, please complete the questions online, print the certificate and present it to your national medical association for review.

## Article Title:  Farm Animal Contact as Risk Factor for Transmission of Bovine-associated *Salmonella* Subtypes

## CME Questions

**1. You are seeing a 40-year-old woman with a 2-day history of diarrhea, fever, and abdominal pain. Initial results from a stool culture demonstrate *Salmonella* species. What should you consider regarding the epidemiology of *Salmonella* infections?**

A. The incidence of *Salmonella* infections has gradually declined over the past 15 years

B. Approximately half of *Salmonella* infections are due to contaminated food

C. Fecal contamination of beef carcasses at the time of slaughter may result in foodborne transmission of *Salmonella*

D. Milk is an even more important means of transmission of *Salmonella* than is meat

**2. Which of the following statements regarding characteristics of cases and controls in the current study is most accurate?**

A. The average age of patients was slightly over 30 years

B. There were as many cases with bovine-associated *Salmonella* as there were controls with non–bovine-associated *Salmonella*

C. The most common serovar among cases was Enteritidis

D. The most common serovar among controls was Typhimurium

**3. You take this patient’s history for possible exposure to *Salmonella*. Which of the following factors was most significantly associated with bovine-associated *Salmonella*?**

A. Consuming undercooked ground beef

B. Contact with farm animals

C. Drinking unpasteurized milk

D. International travel

**4. The patient confirms that she has had some exposure to animals. Which of the following factors was most important in promoting an increased risk for bovine-associated *Salmonella* in the current study?**

A. Attendance at a petting zoo or animal fair

B. Contact with animal manure

C. Working in veterinarian’s office

D. Exposure to cows specifically during the past 5 days

## Activity Evaluation

**Table:** 

**1. The activity supported the learning objectives.**				
Strongly Disagree				Strongly Agree
1	2	3	4	5
**2. The material was organized clearly for learning to occur.**				
Strongly Disagree				Strongly Agree
1	2	3	4	5
**3. The content learned from this activity will impact my practice.**				
Strongly Disagree				Strongly Agree
1	2	3	4	5
**4. The activity was presented objectively and free of commercial bias.**				
Strongly Disagree				Strongly Agree
1	2	3	4	5
